# Supplementary material for: Antibody Repertoire Analysis of Tumor-Infiltrating B Cells Reveals Distinct Signatures and Distributions Across Tissues
Source: Front Immunol. 2021 Jul 19;12:705381. doi: 10.3389/fimmu.2021.705381 (PMC8327180; doi:10.3389/fimmu.2021.705381)
Supplement: Supplementary file 1 [file DataSheet_1.docx]

**Supplementary**

| \| PCR1-FW \| GLUE + 5' Gene specific region \| \| --- \| --- \| \| m-VH-glue-Fw1 \| CCC TCC TTT AAT TCC CGA KGT RMA GCT TCA GGA GTC \| \| m-VH-glue-Fw2 \| CCC TCC TTT AAT TCC CGA GGT BCA GCT BCA GCA GTC \| \| m-VH-glue-Fw3 \| CCC TCC TTT AAT TCC CCA GGT GCA GCT GAA GSA STC \| \| m-VH-glue-Fw4 \| CCC TCC TTT AAT TCC CGA GGT CCA RCT GCA ACA RTC \| \| m-VH-glue-Fw5 \| CCC TCC TTT AAT TCC CCA GGT YCA GCT BCA GCA RTC \| \| m-VH-glue-Fw6 \| CCC TCC TTT AAT TCC CCA GGT YCA RCT GCA GCA GTC \| \| m-VH-glue-Fw7 \| CCC TCC TTT AAT TCC CCA GGT CCA CGT GAA GCA GTC \| \| m-VH-glue-Fw8 \| CCC TCC TTT AAT TCC CGA GGT GAA SST GGT GGA ATC \| \| m-VH-glue-Fw9 \| CCC TCC TTT AAT TCC CGA VGT GAW GYT GGT GGA GTC \| \| m-VH-glue-Fw10 \| CCC TCC TTT AAT TCC CGA GGT GCA GSK GGT GGA GTC \| \| m-VH-glue-Fw11 \| CCC TCC TTT AAT TCC CGA KGT GCA MCT GGT GGA GTC \| \| m-VH-glue-Fw12 \| CCC TCC TTT AAT TCC CGA GGT GAA GCT GAT GGA RTC \| \| m-VH-glue-Fw13 \| CCC TCC TTT AAT TCC CGA GGT GCA RCT TGT TGA GTC \| \| m-VH-glue-Fw14 \| CCC TCC TTT AAT TCC CGA RGT RAA GCT TCT CGA GTC \| \| m-VH-glue-Fw15 \| CCC TCC TTT AAT TCC CGA AGT GAA RST TGA GGA GTC \| \| m-VH-glue-Fw16 \| CCC TCC TTT AAT TCC CCA GGT TAC TCT RAA AGW GTS TG \| \| m-VH-glue-Fw17 \| CCC TCC TTT AAT TCC CCA GGT CCA ACT VCA GCA RCC \| \| m-VH-glue-Fw18 \| CCC TCC TTT AAT TCC CGA TGT GAA CTT GGA AGT GTC \| \| m-VH-glue-Fw19 \| CCC TCC TTT AAT TCC CGA GGT GAA GGT CAT CGA GTC \| \| PCR1- REV \| GLUE + 3' Gene specific region \| \| m-IgMC-BC-glue-REV \| GAG GAG AGA GAG AGA G CG AGG GGG AAG ACA TTT GGG \| \| m-IgGall-BC-glue-REV \| GAG GAG AGA GAG AGA G CC ARK GGA TAG ACH GAT GGG \| \| PCR2-FW \|  \| \| PE-IgALL-Univ-FW \| AAT GAT ACG GCG ACC ACC GAG ATC TAC ACT CTT TCC CTA CAC GAC GCT CTT CCG ATC TNN NNC CCT CCT TTA ATT CCC \| \| PCR2-REV \|  \| \| YW23X_(reverse complement) PE-Idx-REV \| CAA GCA GAA GAC GGC ATA CGA GAT **NNN NNN** GTG ACT GGA GTT CAG ACG TGT GCT CTT CCG ATC TNN NNG AGG AGA GAG AGA GAG \| |
| --- | --- | --- | --- | --- | --- | --- | --- | --- | --- | --- | --- | --- | --- | --- | --- | --- | --- | --- | --- | --- | --- | --- | --- | --- | --- | --- | --- | --- | --- | --- | --- | --- | --- | --- | --- | --- | --- | --- | --- | --- | --- | --- | --- | --- | --- | --- | --- | --- | --- | --- | --- | --- | --- | --- |
| **Table S1.** Primers used for primer extension BCR-Seq. PCR1 primers – FW: V_H_ gene-specific seqeunce (red) and GLUE for primer extension PCR (ligh blue); REV (reverse complement): GLUE (ligh blue), CH1 gene specific sequence (red). PCR2 primers – FW: GLUE for primer extentsion annealing (ligh blue), required diversity region (green), Universal trueSeq Illumina adaptor (purple); REV(reverse complement): GLUE (light blue), required diversity region (green) + T, Universal trueSeq Illumina adaptor. |
|  |
| \| **Mouse** \| **Tissue** \| **Total raw reads** \| **Productive reads** \| **Unique reads** \| **Shared in dupli­cated** \| **Total reads**  **after filtration** \| \| --- \| --- \| --- \| --- \| --- \| --- \| --- \| \| **M1** \| **Tumor** \| 3,013,522 \| 1,545,089 \| 559,089 \| 48,099 \| 1,318,869 \| \| 2,799,957 \| 1,584,532 \| 575,357 \| \| **DLN** \| 6,410,141 \| 4,383,941 \| 2,265,644 \| 92,451 \| 2,366,804 \| \| 1,222,344 \| 837,088 \| 470,405 \| \| **BM** \| 5,311,991 \| 3,838,182 \| 1,803,273 \| 184,023 \| 3,001,259 \| \| 2,764,938 \| 1,989,530 \| 1,051,214 \| \| **PB** \| 819,573 \| 572,017 \| 413,563 \| 25,247 \| 198,512 \| \| 341,713 \| 239,777 \| 183,529 \| \| **M2** \| **Tumor** \| 2,159,444 \| 1236417 \| 271922 \| 21708 \| 1,216,678 \| \| 2,356,565 \| 1495127 \| 330478 \| \| **PB** \| 478,136 \| 331,650 \| 240,726 \| 15,944 \| 103,160 \| \| 184,796 \| 127,058 \| 104,631 \| \| **DLN** \| 764,428 \| 500940 \| 342,847 \| 28,840 \| 233,689 \| \| 887,901 \| 590451 \| 412,871 \| \| **BM** \| 1,362,027 \| 952,835 \| 526,383 \| 81,425 \| 875,626 \| \| 1,559,131 \| 1,104,857 \| 587,970 \| \| **M3** \| **Tumor** \| 1,044,197 \| 648,208 \| 220,884 \| 28,072 \| 680,448 \| \| 758,064 \| 469,970 \| 167,227 \| \| **PB** \| 467,524 \| 307,216 \| 221,817 \| 30,823 \| 183,961 \| \| 504,876 \| 331,817 \| 236,430 \| \| **DLN** \| 1,006,525 \| 587,993 \| 271,983 \| 13,905 \| 367,083 \| \| 1,283,181 \| 743,753 \| 307,615 \| \| **BM** \| 725,273 \| 484,223 \| 277,649 \| 51,213 \| 653,939 \| \| 1,418,660 \| 965,328 \| 500,111 \| \| **M4** \| **Tumor** \| 172,148 \| 121,997 \| 30,847 \| 7,995 \| 228,652 \| \| 275,603 \| 190,847 \| 43,671 \| \| **PB** \| 927,959 \| 633,340 \| 284,306 \| 40,217 \| 439,785 \| \| 391,222 \| 268,607 \| 142,080 \| \| **DLN** \| 1,447,467 \| 873,367 \| 268,364 \| 36849 \| 926,849 \| \| 1,248,512 \| 749,077 \| 249,827 \| \| **BM** \| 90,633 \| 65,017 \| 26,005 \| 4168 \| 76680 \| \| 99,345 \| 69,266 \| 26,360 \| \| **N1** \| **LN** \| 1,182,417 \| 735,776 \| 500,713 \| 11722 \| 45313 \| \| 156147 \| 91,531 \| 82725 \| \| **BM** \| 1602662 \| 1084211 \| 755729 \| 42223 \| 356409 \| \| 459945 \| 307263 \| 238084 \| \| **Blood** \| 1559966 \| 1102181 \| 612507 \| 42566 \| 510345 \| \| 370609 \| 242924 \| 161998 \| \| **N2** \| **LN** \| 640,750 \| 414,668 \| 337,538 \| 22788 \| 58056 \| \| 564218 \| 360,448 \| 300636 \| \| **BM** \| 434917 \| 300192 \| 234853 \| 17803 \| 140258 \| \| 485689 \| 334271 \| 263834 \| \| **Blood** \| 393680 \| 263335 \| 193244 \| 23536 \| 144656 \| \| 383011 \| 250325 \| 187367 \| |
| **Table S2. Summary of BCR-Seq data.** *Total raw reads*: reads obtained from the fastq files following paired-end alignment; *productive reads:* reads that were successfully aligned to the germline reference in IMGT; *exist in both duplicates:* unique V_H_ sequences that were shared between the technical duplicates; *total reads after filtration;* V_H_ sequences that were shared between technical duplicates (non-unique) and passed the filtration process.  T - treated, N - naïve. |

| \| **Mouse #** \| No. of clones \| \| \| \| \| --- \| --- \| --- \| --- \| --- \| \|  \| **Tumor** \| **DLN** \| **Blood** \| **Bone marrow** \| \| M1 \| 1897 \| 9212 \| 4813 \| 23,597 \| \| M2 \| 785 \| 6418 \| 3621 \| 15790 \| \| M3 \| 1992 \| 934 \| 6766 \| 8546 \| \| M4 \| 352 \| 2289 \| 7025 \| 533 \| \| Average \| 1256.5 \| 4713.25 \| 5556.25 \| 12116.5 \| |
| --- | --- | --- | --- | --- | --- | --- | --- | --- | --- | --- | --- | --- | --- | --- | --- | --- | --- | --- | --- | --- | --- | --- | --- | --- | --- | --- | --- | --- | --- | --- | --- | --- | --- | --- | --- |
| **Table S3.** **Number of total B cell clones in the different tissues.** |

| **Mouse #** | No. of TIL-B common clones | | |
| --- | --- | --- | --- |
|  | **DLN** | **Blood** | **Bone marrow** |
| M1 | 102 | 94 | 184 |
| M2 | 40 | 44 | 54 |
| M3 | 27 | 254 | 73 |
| M4 | 8 | 56 | 2 |
| **Table S4. Number of common TIL-B clones.** The number of TIL-B common clones represents the clones that appear in the tumor of each mouse and in either the DLN, Blood or Bone marrow of the same mouse. | | | |

| **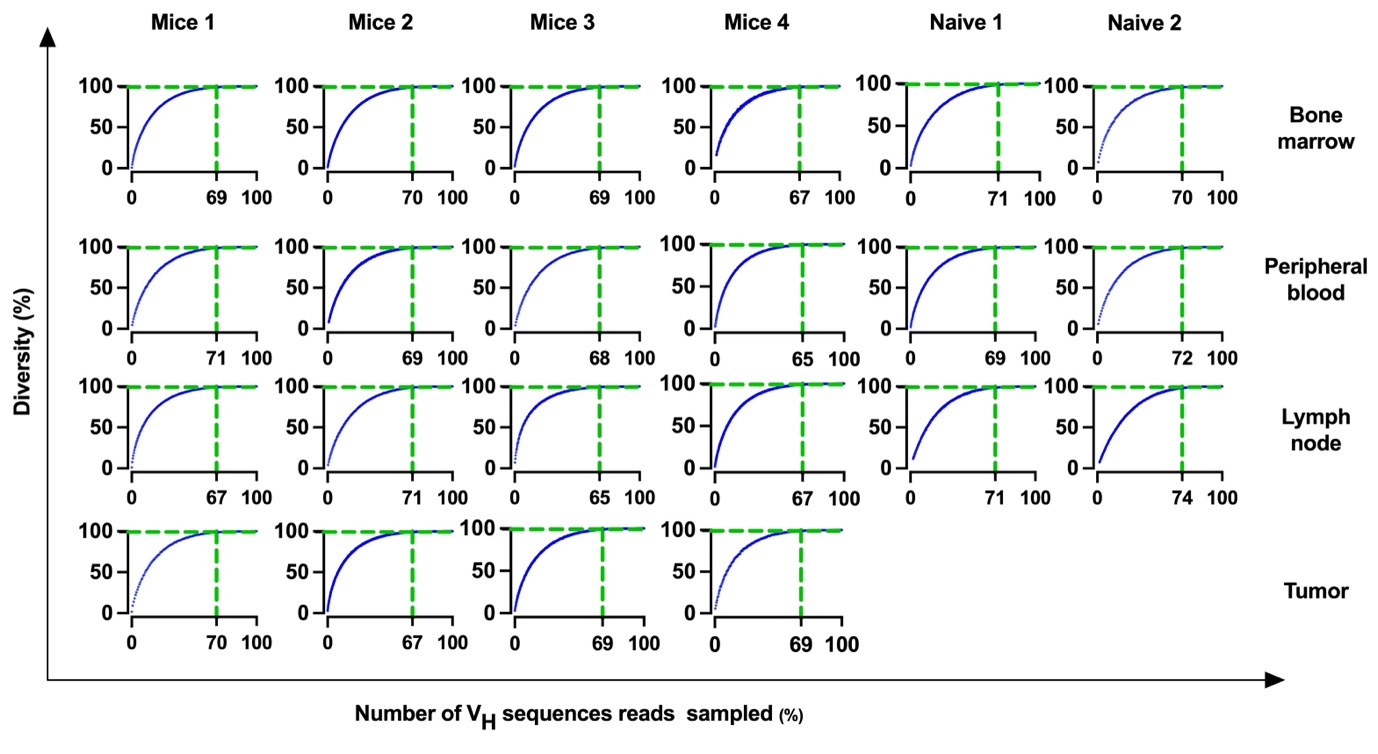** |
| --- |
| **Figure S1:** **Estimation of sequencing depth as a function of sampling.** Rarefaction curves represent sequence diversity (= number of unique V_H_ sequences, Y-axis) as a function of the number of V_H_ sequences reads sampled (X-axis) in each iteration (random sampling). As the curve saturates, an increase in sample size does not affect sample diversity; therefore, the sequencing depth is adequate. The vertical dashed line indicates the percent of sequences reads in which unique V_H_ sequence diversity reaches 99%. X-axis units represent the fraction of V_H_ sequences reads in each sampling iteration, out of the total number of V_H_ sequences reads; Y-axis represents the fraction of unique V_H_ seqeunces in each sampling iteration out of the total unique V_H_ sequences. |

| 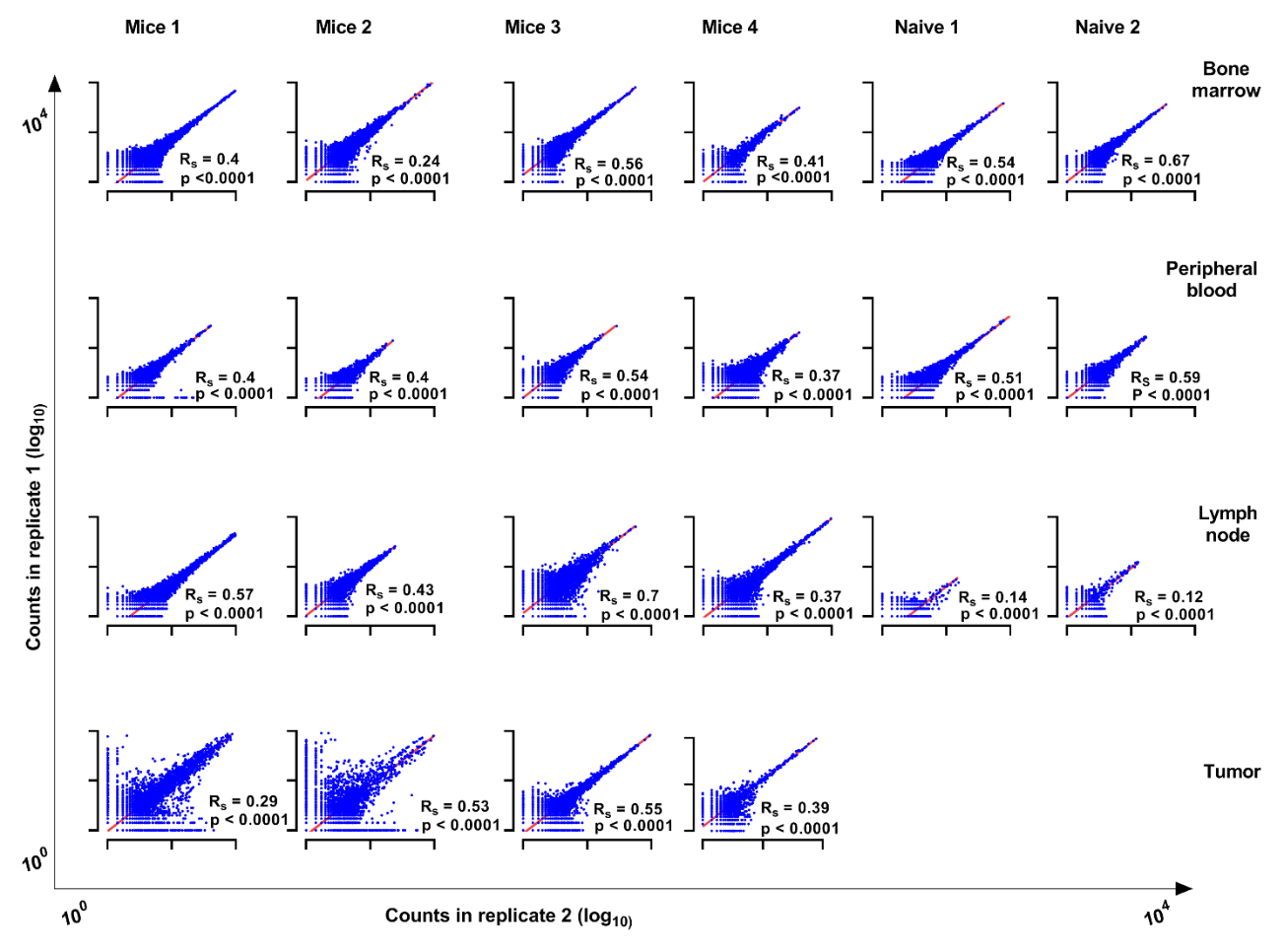 |
| --- |
| **Figure S2:** **Spearman's rank correlation of BCR-Seq duplicates**. Each dot represents a unique V_H_ sequence. The X and Y axes indicate the number of read counts for each V_H_ in the duplicates (logarithmic scale). R_S_ - Spearman's correlation coefficient, p – two tailed was considered significant if <0.05. |

| 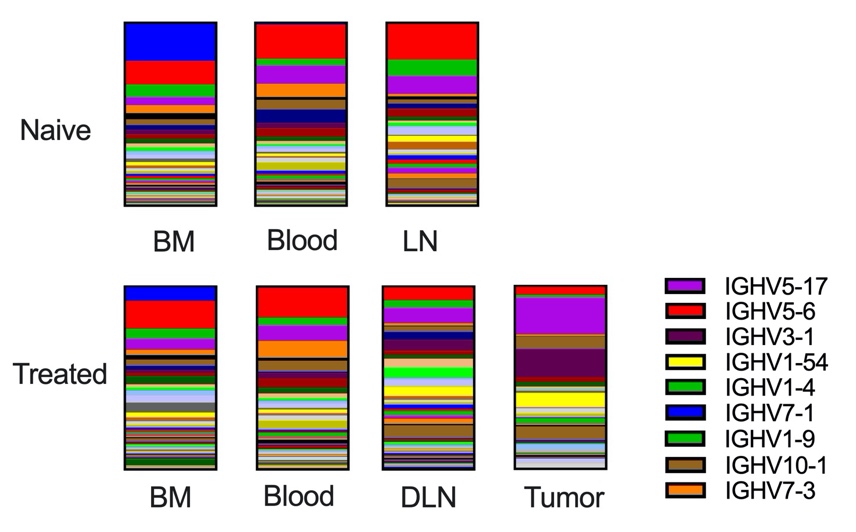 |
| --- |
| **Figure S3:** **Stacked bar plot of V gene usage in each compartment.** The proportion of each section in the stacked bar represents the frequency of V genes in each compartment. Selected V gene are shown in the legend. |

| 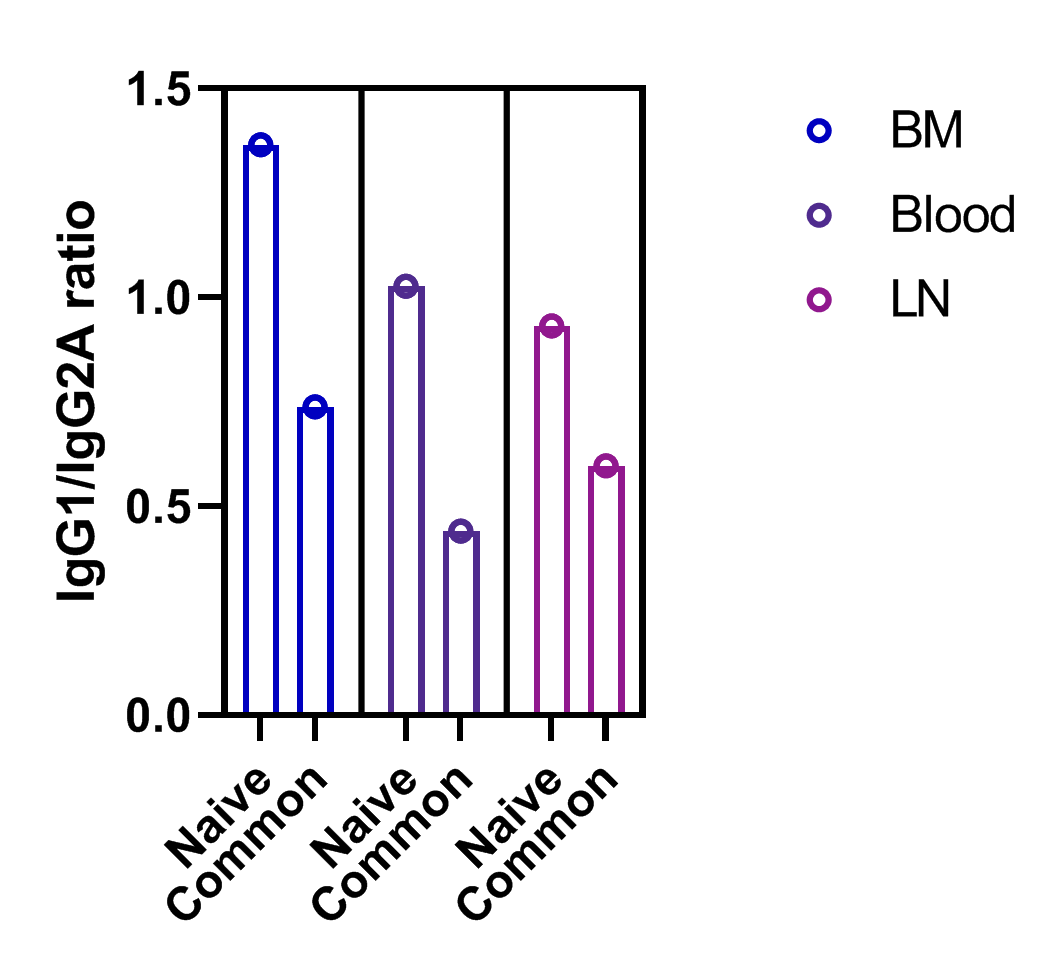 |
| --- |
| **Figure S4:** **Calculated ratio between IgG1 and IgG2A subclasses.** The ratio was calculated by dividing the relative frequency of antibodies with the IgG1 subclass by that with the IgG2A subclass as encoded by B cells in 3 tissue types from naïve mice and common clones to the tumor and the corresponding tissues in the treated mice (i.e., common to the tumor and bone marrow, blood or DLNs). |
